# Observing the invisible: X‐ray CT for plant–microbe interactions

**DOI:** 10.1111/nph.71252

**Published:** 2026-05-10

**Authors:** Eric C. Pereira, Chris A. Bell

**Affiliations:** ^1^ Research School of Biology The Australian National University Canberra ACT 2601 Australia; ^2^ School of Biology, Faculty of Biological Sciences University of Leeds Leeds LS2 9JT UK

**Keywords:** 3D imaging, digital rhizosphere, plant pathology, plant–microbe interactions, rhizosphere, root system architecture, X‐ray computed tomography

## Abstract

Plant–microbe interactions are inherently spatial, yet the physical structure of the soil and rhizosphere is rarely treated as a mechanistic variable in experimental design. X‐ray computed tomography (X‐ray CT) enables nondestructive, three‐dimensional, and time‐resolved imaging of intact root–soil systems, providing direct access to the structural context in which plant–microbe interactions occur. Rather than a secondary imaging technique, X‐ray CT can offer a wealth of data as a primary experimental platform for future plant–microbe research. Here, we highlight key structural traits that X‐ray CT can quantify and discuss how they may shape microbial behaviour, plant immune responses, and disease outcomes. We expand on how X‐ray CT could be employed in future to provide a framework to disentangle direct microbial effects from indirect, structure‐mediated feedbacks. For breeding and management, it could enable selection for root traits and soil practices that engineer favourable microhabitats rather than targeting organisms in isolation. Despite this potential, broader adoption will require overcoming current limitations related to access to instrumentation, analytical expertise, and the integration of structural data with biological measurements. Overall, we suggest that resolving these issues will enable the integration of X‐ray CT‐derived structure with molecular, microbiome, and modelling approaches to enable the development of digital rhizospheres, offering a pathway from descriptive observations to predictive, structure‐aware *in silico* frameworks in plant–microbe research.

## Graphical abstract

Utility of X‐ray computed tomography (X‐ray CT) for visualising belowground plant interactions between multiple spatial scales and focal planes.

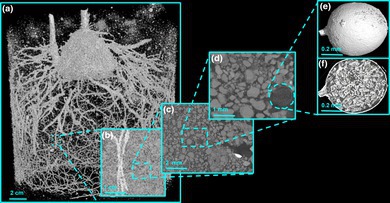

## Introduction

Plant–microbe interactions can influence the development, nutrition, immunity, and stress tolerance of the plant host, and shape the microbiome not only through genetics and biochemical signalling but also through physical structures (Downie *et al*., 2015; Roose *et al*., 2016; Gregory *et al*., 2022). Many of the most influential interactions occur in the soil, where roots grow within an opaque, heterogeneous matrix of minerals, aggregates, and pores that govern water, oxygen, and microbial dispersion (Moradi *et al*., 2011; Helliwell *et al*., 2017). Visualising interactions within the soil, in roots, or at the interface between the two poses a unique experimental challenge, often leaving structural contexts poorly quantified and treated implicitly in plant–microbe studies. Over the last decade, an expanding toolbox of imaging and sensing technologies has begun to address this challenge, enabling the capture of aspects of root architecture, water distribution, and metabolite patterning with minimal disturbance. These advances complement genetic and biochemical approaches by revealing how the physical environment contributes to shaping plant–microbe interactions (Moradi *et al*., 2011; Downie *et al*., 2012, 2015; Warner *et al*., 2014; Lohse *et al*., 2021; Gregory *et al*., 2022; Ahkami *et al*., 2024). Alongside these advances, X‐ray computed tomography (X‐ray CT) has emerged as a highly versatile tool for three‐dimensional imaging of intact root–soil systems. Originally developed in soil physics and geosciences, X‐ray CT now resolves roots, soil structure, and pore networks *in situ* (Garbout *et al*., 2013; Helliwell *et al*., 2014; Ghosh *et al*., 2023), enabling direct quantification of root and soil metrics in natural substrates and supporting automated pipelines for three‐dimensional (3D) root phenotyping (Keyes *et al*., 2013; Rogers *et al*., 2016; Mairhofer *et al*., 2015; Teramoto *et al*., 2020).

In this Viewpoint, we propose that X‐ray CT should be integrated within plant–microbe research rather than a secondary imaging technique. We highlight key structural traits that it can quantify, show how these traits inform plant immune responses, microbiome assembly, and disease outcomes, and outline how X‐ray CT can serve as the foundation of integrated ‘digital rhizospheres’ linking physical structure to biological function.

## X‐ray CT as a structural backbone for plant–microbe research

**Fig. 1:**
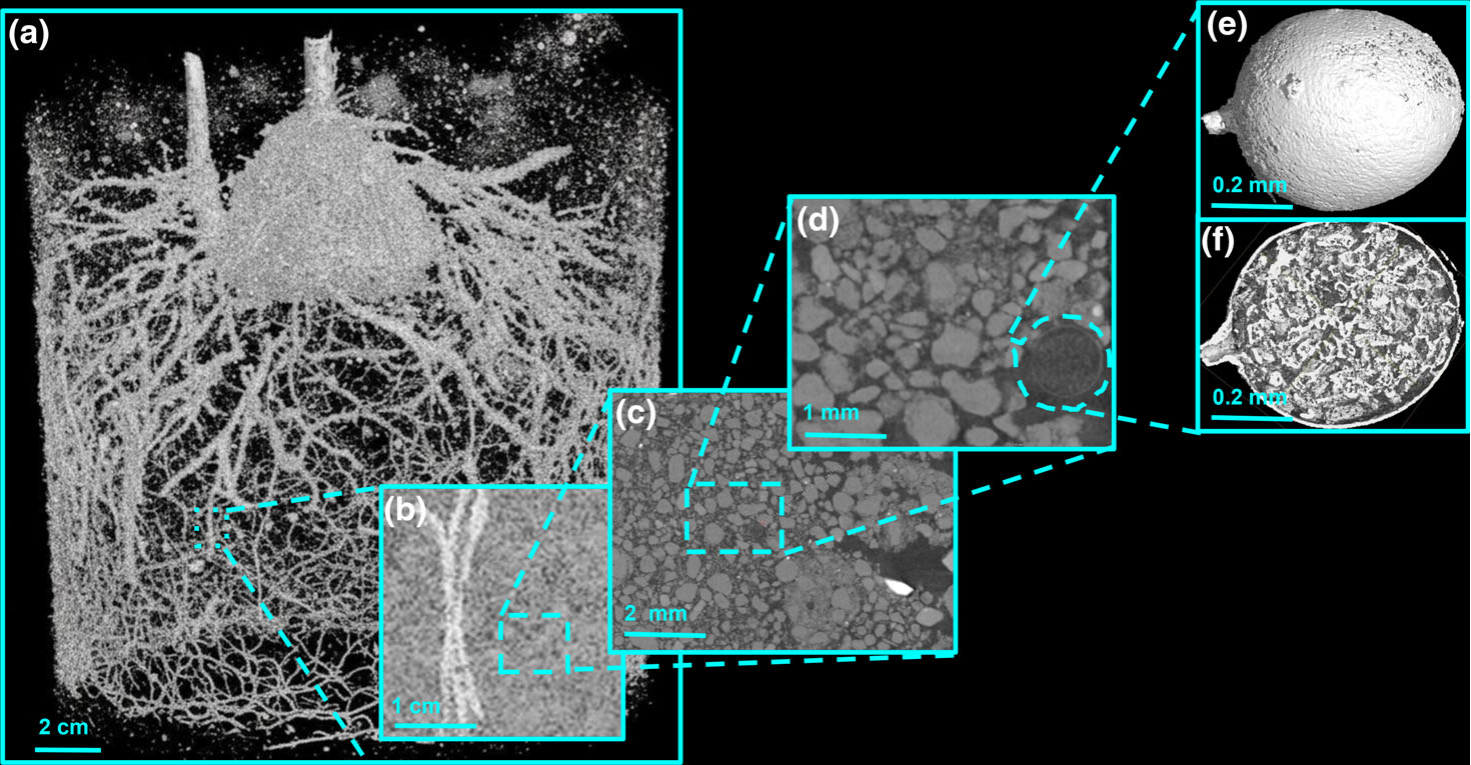
Utility of X‐ray computer tomography (X‐ray CT) for visualising belowground plant interactions across multiple spatial scales and focal planes. (a) X‐ray CT image of a 3‐wk‐old *Solanum tuberosum* (potato) grown in a 2‐l soil–sand medium, showing the developing root system emerging from the seed tuber (top centre). Bar, 2 cm. (b) Higher magnification scan of the same pot, highlighting root structures within the surrounding soil matrix. Bar, 1 cm. (c) Higher resolution inset of the same region, illustrating improved separation of soil particles and pore spaces; pores appear as dark regions surrounding grey soil particles. Bar, 2 mm. (d) Further magnified inset, highlighting a single *Globodera pallida* cyst (dashed outline) within the soil matrix. Bar, 1 mm. (e) External view of the single cyst observed in (d). (f) X‐ray CT segmentation and cross‐sectional analysis of the cyst visualised in (e) to estimate the number of enclosed eggs. Bar, 0.2 mm. Dashed boxes indicate the regions enlarged in subsequent panels. Insets may visualise different focal planes within the same scanned volume, to present features hidden behind soil or roots, that is the nematode cyst. Greyscale reflects relative structural densities within each panel. Samples were characterised using a GE Phoenix micro‐CT system (a) and GE Nanotom M X‐ray CT machine (b–f) (GE Measurement and Control Solutions, Germany). The v|tome|x M was set at a voltage of 65 kV and a current of 300 μA to optimise contrast between background soil and objects of interest. The ‘Fast Scan option’ achieved a voxel resolution of 1.60 μm. A total of 1078 projection images were taken per scan at 200 m s^−1^ per image. Total scan duration was 30 min for the whole‐pot scan (a–d) and 10 min for the high‐resolution cyst scans (e, f). The images were reconstructed using Phoenix datos| × 2 rec reconstruction software, combining the scans into a single three‐dimensional volume representing the entire sample (Pereira *et al*., 2025).

The defining strength of X‐ray CT is its ability to describe structure as an integrated 3D system that can be measured over experimental timelines. For plant–microbe research, three domains that are quantifiable by X‐ray CT are particularly important: root system architecture (RSA), soil pore and aggregate structure, and the root–soil interface, all of which may contain interacting organisms (Fig. 1).

### Roots in soil: architecture as habitat engineering

RSA determines where and how plants interact with soil microbiota. Root length, branching density, and gradients in exudate, oxygen, and nutrient depth distributions define colonisation opportunities for both beneficial and pathogenic organisms (Rogers *et al*., 2016), beyond what is possible with transparent media and destructive excavation (Mairhofer *et al*., 2013; Teramoto *et al*., 2020). These time‐resolved measurements reveal roots as active engineers of their physical habitat, displacing soil particles, opening or closing pores, and reshaping connectivity as they grow (Helliwell *et al*., 2017). For microbes, these changes will not be incidental and likely regulate access to root surfaces, dispersal routes through soil, and the persistence of local niches. From a plant–microbe perspective, RSA is not an abstract plant trait independent of soil, therefore will have profound effects on the microorganism's natural habitat. Future research should utilise methods that can combine RSA, soil metrics, and the interactions between the two to enable direct links between root traits, rhizosphere structure, and microbial outcomes. Additionally, plant–microbe interactions are often studied as a single root system interacting with a single symbiont (Hartman *et al*., 2023). By utilising methods such as X‐ray CT, we could begin to untangle the effects of multiple hosts and potentially multiple symbionts, be they mutualistic or parasitic or both, and their concomitant effects.

### Soil architecture: pores, aggregates, and connectivity

Soil structure regulates microbial life by controlling water retention, gas diffusion, and physical accessibility. Pore‐size distributions determine which organisms can move where, the location of anoxic microsites, and how rapidly substrates are replenished, while aggregate architecture creates refugia from predation and disturbance (Baveye *et al*., 2018). Pore networks can be characterised via X‐ray CT by size, connectivity, and tortuosity, and aggregates can be defined by size, shape, and contact relationships (Garbout *et al*., 2013; Budhathoki *et al*., 2022) (Fig. 1). Unlike bulk descriptors such as texture or bulk density, these metrics describe the actual microhabitats experienced by microbes. For plant–microbe research, this distinction is critical. Two soils with identical texture can host very different microbial dynamics if their pore networks differ in connectivity or their aggregates differ in stability (Roose *et al*., 2016; Juyal *et al*., 2019). Additional research is needed to broaden this area of investigation.

### The root–soil interface: where interactions happen

The rhizosphere is a physically distinct, continuously remodelled zone at the root–soil interface. As roots grow, they deform surrounding soil, which generates biopores and compresses aggregates, thereby altering pore connectivity within millimetres of the root surface (Helliwell *et al*., 2017; Koebernick *et al*., 2018; Lucas *et al*., 2019; Ghosh *et al*., 2023). These changes determine where exudates accumulate, where oxygen is depleted, and where microbes can physically access the root. At finer scales, synchrotron CT has revealed that even root hairs create distinct structural zones in the surrounding soil, altering pore geometry at scales directly relevant to microbial colonisation (Koebernick *et al*., 2017). X‐ray CT is currently the most practical approach for quantifying this interface in three dimensions in intact soil. Crucially, because scanning is nondestructive, the same sample can be imaged repeatedly, enabling direct tracking through time. Together, roots, soil architecture, and interfaces form a coupled structural system that governs microbial ecology and plant health. X‐ray CT captures this system at a resolution and throughput that enable a whole‐pot scan, achievable in 30 min, for example, that no other single technique currently matches for opaque soils. The current limitation is the limited application of X‐ray CT, restricting time‐resolved spatial insights into plant systems.

## Applications within plant–microbe interactions

### Pathogen dynamics and spatial diagnostics

A key biological insight from structure‐aware studies is that pathogen abundance alone does not predict disease severity, as the structural context in which pathogens encounter roots is equally important. In damping‐off caused by *Rhizoctonia solani*, coupling X‐ray CT with quantitative reverse transcription polymerase chain reaction revealed that 3D patterns of root damage did not scale linearly with pathogen abundance, demonstrating that disease outcomes were shaped by the interaction between pathogen pressure and local root–soil structure (Sturrock *et al*., 2015). This finding would not have emerged from molecular quantification alone, and it challenges purely abundance‐based frameworks for understanding soil‐borne disease. Similar approaches have visualised *Armillaria* infection of woody roots, linking disease progression to altered root connectivity and soil contact (Zhang *et al*., 2022). X‐ray CT has recently enabled direct, nondestructive quantification of *Globodera pallida* (a plant–parasitic nematode) in soil, showing strong concordance with conventional extraction methods while preserving depth and spatial context (Pereira *et al*., 2025) (Fig. 1). These studies point towards structure‐aware diagnostics that integrate pathogen abundance with habitat characteristics and propose utilisation of X‐ray CT in avenues beyond research, such as border control soil screening. Academically, assessing RSA in the context of dynamic biotic challenges remains an underdeveloped opportunity; for example, longitudinal X‐ray CT imaging of root‐knot nematode infections could capture how gall formation perturbs systemic root architecture over time, providing a true four‐dimensional view of host–pathogen interactions. Indeed, this approach could be extended to other gall‐forming root symbioses and pathologies that similarly, or not as the resultant data may tell us, reshape local and whole‐root system structure. Furthermore, combining fluorescent microscopy approaches will enable the tracking of reporters with root architecture and symbiont development, offering a wealth of architectural and molecular data.

### Beneficial symbiosis and root‐driven soil restructuring

Beneficial plant–microbe interactions, including arbuscular mycorrhizal (AM) fungal symbioses, can modify soil structure, but direct evidence in intact soils has been limited by destructive sampling. X‐ray CT overcomes this constraint by enabling nondestructive, 3D quantification of how roots and associated microbes reshape soil architecture *in situ*. CT‐based studies show that manipulation of soil microbial communities or AM fungal colonisation alters aggregate formation and pore connectivity, demonstrating that beneficial symbioses can actively engineer soil structure at the scale of intact soil cores (Martin *et al*., 2012; Zheng *et al*., 2023). Further studies have utilised X‐ray CT not only to visualise AM fungi but also to perform elemental mapping, showing fungal localisation within soils relative to nutrient profiles and the wider soil microbiome (Keyes *et al*., 2022).

More broadly, correlative approaches combining X‐ray CT with techniques such as NanoSIMS and FIB‐SEM have begun to trace the fate of root‐derived organic matter within intact rhizosphere structures, revealing how roots and associated biota co‐create the physical environments that govern nutrient cycling (Mueller *et al*., 2024). From a plant–microbe perspective, this indicates that some benefits attributed to symbioses, such as improved nutrient acquisition or stress tolerance, may arise not only from direct molecular interactions but also from indirect, structure‐mediated effects that can now be measured explicitly.

### Soil microstructure and emergent microbial behaviour

A central insight from soil microbiology is that microbial activity is governed by microhabitats rather than bulk soil properties (Tecon & Or, 2017; Baveye *et al*., 2018). Structure is not merely context, as it actively drives microbial function. Li *et al*. (2024) demonstrated this directly by combining X‐ray CT‐characterised pore structure with ^13^C stable isotope probing, revealing that large and small soil pores harbour distinct microbial communities with fundamentally different metabolic strategies and carbon‐processing pathways. Similarly, Kravchenko *et al*. (2019) showed that plant‐stimulated pore formation in the 30–150 μm range determines whether new carbon inputs are stabilised or lost to the atmosphere, linking physical structure to ecosystem‐level carbon cycling. These findings establish that X‐ray CT‐quantifiable structural shapes not only where microbes are but also what they do. Supporting this principle, X‐ray CT‐based microcosm studies show that pore connectivity and solid‐pore interface area strongly influence microbial dispersal and colonisation, even under identical bulk soil conditions (Juyal *et al*., 2019). Similarly, microbiome profiling of soil microcosms with contrasting pore characteristics has shown that both bacterial and fungal community structure vary with pore size and moisture content (Benucci *et al*., 2023), reinforcing the principle that X‐ray CT‐quantifiable structure shapes microbial community assembly. Combined X‐ray CT and microscopy approaches further demonstrate that specific pore classes identified in X‐ray CT volumes are preferentially occupied by microbes, confirming that microbial spatial organisation can be predicted from measurable structural features (Negassa *et al*., 2015). Obtaining similar insights for a broader range of microorganisms, or even animals who reside in the soil, will have profound impact on our awareness of the impact of soil properties on the use of mutualists, the control of pathogens, and negating abiotic stresses.

Although many of these studies do not explicitly include plant variables, they establish a critical principle for plant–microbe research: microbial processes become predictable when structural context is quantified at the appropriate scale.

## Strengths, limitations, and scale trade‐offs

X‐ray CT's major strength is its capacity to repeatedly image intact plant–soil systems, enabling temporal 3D tracking of the subject. Each sample serves as its own temporal reference, reducing variability and enabling direct quantification of dynamic processes. Radiation exposure must be considered in longitudinal work, yet multiple studies show that carefully managed micro‐CT doses do not measurably affect plant growth or soil microbial activity (Zappalà *et al*., 2013). If further studies show these effects to be context‐dependent, it may require case‐by‐case optimisation of scan parameters, frequency, and total dose according to the biological system and experimental design. A correlative workflow developed by Lippold *et al*. (2023) demonstrates this approach in practice, using CT‐defined root–soil geometry to guide targeted chemical mapping of nutrient gradients in the rhizosphere, illustrating how structural data can direct and anchor molecular analyses. X‐ray CT is therefore suitable for time‐resolved plant–microbe experiments when the dose is treated as an experimental parameter.

X‐ray CT datasets provide rich structural descriptors whose value for plant–microbe interactions lies in their interpretation as habitat variables: pore connectivity shapes oxygen supply and microbial dispersal, aggregate architecture defines refuges and reaction sites, and root–soil contact governs the spatial distribution of exudates and signals (Baveye *et al*., 2018) (Fig. 1). A key limitation, however, is that X‐ray CT‐derived structure alone cannot explain biological outcomes without integration with organismal and pathogen biology.

Equally important is clarity about what X‐ray CT does not resolve. Individual microbial cells can be below their resolution and contrast limits; X‐ray CT would image the habitat, not the microbes themselves. This limitation is negated somewhat if we are able to build up strong predictors of microbe habitats and link with additional methods, outlined later (see Data integration and the rise of digital rhizospheres). Resolution and sample size are tightly coupled in benchtop systems that resolve roots and macropores, whereas synchrotron CT can reach submicrometre scales at the cost of throughput and accessibility (Keyes *et al*., 2013). Effective experimental design, therefore, requires matching resolution and sample volume to the biological processes of interest. Future technical developments, including higher resolution synchrotron imaging, contrast agents, and correlative multimodal workflows, as well as fluorescent labelling of soil symbionts, may extend detection limits to individual microorganisms in future.

A further limitation is that X‐ray CT requires specialist equipment, analytical expertise, and substantial computational capacity. These barriers are most readily overcome through collaboration. Stronger links among plant pathologists, soil physicists, and X‐ray CT specialists, and the extension to computer scientists developing automated segmentation, registration, and structural analytics, would accelerate progress and support the development of integrated digital rhizospheres (see Data integration and the rise of digital rhizospheres). Such interdisciplinary partnerships are essential for scaling X‐ray CT from a technical capability to a mainstream platform for plant–microbe research.

Currently, several complementary approaches exist for spatially resolved investigation of root–soil systems, each with distinct strengths and trade‐offs. Rhizobox and rhizotron systems provide direct visual access to the root–soil interface but are limited to two‐dimensional observations at the container surface and introduce edge effects that alter root behaviour. Transparent soil analogues enable optical imaging of root growth and microbial colonisation in three dimensions, but substitute natural soil structure with artificial matrices that differ in physical and hydraulic properties (Downie *et al*., 2012). Equipment and methodologies such as Magnetic Resonance Imaging provide excellent contrast for water distribution and can track root development nondestructively, but typically achieve lower spatial resolution for solid‐phase soil structure than X‐ray CT and are sensitive to soil type, with some substrates producing imaging artefacts (van Dusschoten *et al*., 2016). Neutron imaging excels at resolving water content and root–water relations at high temporal resolution, although it requires access to a reactor or spallation source, offers limited contrast between mineral phases, and works best at low soil moisture levels (Cai *et al*., 2022). Synchrotron CT achieves submicrometre resolution and can incorporate elemental or chemical contrast through spectroscopic modes but is constrained to small sample volumes and limited beam‐time availability (Keyes *et al*., 2013). Within this landscape, benchtop X‐ray CT occupies a distinctive niche, as it enables repeated, nondestructive, 3D imaging of intact opaque ‘real’ soil at resolutions sufficient to resolve roots, macropores, and mesofauna, with a throughput and accessibility that support routine experimental integration. We propose that if utilised appropriately, these capabilities of X‐ray CT allow it to yield insights into broad biological questions. Employing methods such as X‐ray CT will help us to generate data that are more applicable to the scenario we are researching without creating additional artificial systems that each have their own pitfalls and edge further away from the question in study. For example, control of pathogens such as plant–parasitic nematodes can be achieved by nematicides or biological agents such as *Bacillus;* however, the response of each to different soil types, or the effects of different soil organisms on the efficacy of these different approaches, is unknown. The application of X‐ray CT could provide major insights into the dynamics of such approaches in varying real‐world soils to not only provide extra datasets but also optimise and direct the creation of new products with greater transferable efficacy, potentially across vastly different abiotic conditions.

In future, X‐ray CT‐derived structural metrics could be coupled with complementary imaging, such as fluorescence microscopy, hyperspectral imaging, and chemical mapping to generate multimodal plant–microbe data, such as microbial/infection hotspots, root exudation zones, and plant traits. An added bonus of X‐ray CT is the strong potential for AI assistance, particularly regarding data analysis. Current practices scan the roots and then often manually annotate features. AI‐assisted segmentation now outperforms manual approaches for delineating roots, pores, and aggregates in noisy or low‐contrast volumes, reducing analyst bias and enabling high‐throughput processing of large datasets (Smith *et al*., 2022; Phalempin *et al*., 2025). The current bottleneck of limited annotated datasets that cover a low number of soil types will be overcome as the number of available X‐ray CT datasets increases, allowing robust benchmarking and subsequent enhanced reproducibility of studies across analytical pipelines. There will be a pivotal threshold in which the number of available studies enables AI predictions of such a high quality that they reduce the necessity for additional manually annotated datasets.

## Data integration and the rise of digital rhizospheres

The full value of X‐ray CT for plant–microbe research will emerge when structural information is integrated with molecular, microbiome, and modelling approaches, rather than treated as supplementary context. X‐ray CT‐derived architecture can provide the missing spatial scaffold, enabling mechanistic interpretation of biological measurements rather than purely statistical analysis. This integration will enable a transition from descriptive associations to predictive, structure‐aware frameworks, which we can refer to here as digital rhizospheres. If developed systematically, such structure‐aware frameworks have the potential to shift plant–microbe research from purely descriptive associations towards predictive, spatially explicit analyses, a transition that would complement the molecular resolution already achieved through sequencing‐based approaches.

X‐ray CT data are inherently volumetric, but their biological relevance depends on how they are translated into traits. Traditional outputs, such as total root length or mean pore size, are informative but insufficient for plant–microbe questions. For example, what matters biologically are relative spatial relationships, such as distances between roots and pores, connectivity of pore networks near root surfaces, interface areas between aggregates and roots, and the spatial clustering of propagules within defined microhabitats (Roose *et al*., 2016; Helliwell *et al*., 2017). In practice, these traits are derived from a series of image‐processing steps applied to segmented X‐ray CT volumes. Pore connectivity is typically quantified via connected component labelling or percolation analysis on binarised pore networks, yielding metrics such as the proportion of connected pore space and path tortuosity (Schlüter *et al*., 2014). Root–soil contact is measured as the fraction of root surface area directly abutting soil solids vs air‐filled pores, computed from distance maps applied to coregistered root and soil segmentations (Koebernick *et al*., 2018; Phalempin *et al*., 2026). Aggregate metrics, including size distributions, shape factors, and surface roughness, are derived from watershed segmentation of the solid phase (Budhathoki *et al*., 2022). While these approaches are increasingly routine, full standardisation of trait definitions and reporting protocols across laboratories remains an important priority, as we discuss later (see Future Priority 2: ‘Standardise reporting and trait definitions’). Recent advances in segmentation and feature extraction now enable the routine computation of such traits from X‐ray CT volumes. Tools such as RooTrak enable automated segmentation and tracking of root systems within CT volumes (Mairhofer *et al*., 2013), while 4DRoot provides an open‐source pipeline for extracting root length, branching density, and volume across successive scans (Herrero‐Huerta *et al*., 2022). More broadly, machine‐learning‐based approaches have improved robustness across soil types and moisture conditions, reducing manual intervention and enabling high‐throughput analyses (Ghosh *et al*., 2023).

### Linking X‐ray CT‐derived structure to omics and microbiome data

A major opportunity lies in explicitly linking X‐ray CT‐defined structure to molecular and microbiome datasets. X‐ray CT is compatible with downstream DNA, RNA, and metabolite analyses, enabling integrated experimental designs in which structure and biology are measured on the same samples (Zappalà *et al*., 2013). Structural traits derived from X‐ray CT can subsequently be used as predictors in statistical or mechanistic models to explain variation in microbial community composition, functional gene abundance, or metabolite profiles (Li *et al*., 2024). Uniting multiple distinct methods has far‐reaching translations throughout other systems.

When X‐ray CT‐derived structure and biological data are coupled with modelling, we envisage that they will enable the construction of transformative digital rhizospheres, computational representations of specific plant–soil–microbe systems grounded in measured geometry rather than idealised assumptions. Such models can explore how subtle structural changes alter oxygen dynamics, exudate gradients, or the spatial overlap between pathogens and antagonists (Roose *et al*., 2016). Schnepf *et al*. (2022) outlined a multiscale modelling framework that explicitly uses X‐ray CT‐derived pore geometries to bridge molecular, pore, and root‐system scales, providing a template for the kind of structure‐aware modelling that digital rhizospheres will require. This provides the groundwork for larger approaches that could incorporate structural soil descriptors into crop, nutrient, or disease models as state variables.

Crucially, these digital rhizospheres could be iteratively refined through feedback between experiments and models. X‐ray CT‐informed simulations could generate predictions about which structural configurations favour disease suppression, nutrient acquisition, or microbiome stability. Omics integration could then test these predictions, feeding results back into model refinement. Over time, this loop transforms X‐ray CT from a descriptive imaging method into a predictive engine for plant–microbe interactions. By continually optimising, building, and expanding digital datasets, we could then provide AI tools with sufficient data to produce numerous high‐quality models across varying scenarios.

The emergence of digital rhizospheres has practical implications. In plant pathology, it will enable spatially explicit risk assessment, in which pathogen pressure is interpreted in the context of root architecture and soil structure rather than solely by abundance. For microbiome research, it will provide a framework to disentangle direct microbial effects from indirect, structure‐mediated feedbacks. For breeding and management, it will enable selection for root traits and soil practices that engineer favourable microhabitats rather than targeting microbes in isolation.

Despite these capabilities, X‐ray CT has not yet been widely adopted in plant–microbe research, and understanding why is important for charting a realistic path forward. Three principal barriers can be identified. The first is access and cost, as benchtop X‐ray CT systems typically require capital investment. Relatively few plant–microbe research groups currently have routine access. The second is expertise in acquiring, reconstructing, and segmenting X‐ray CT data. This requires skills in physics, image processing, and increasingly machine learning that are not standard training in plant pathology or microbial ecology. The third is cultural distance, because X‐ray CT has developed primarily within soil physics and geoscience communities, whose research questions, publication venues and professional networks have historically had limited overlap with plant–microbe biology. These barriers are not insurmountable. Benchtop systems are becoming more accessible through shared institutional facilities, and open‐source segmentation tools and training resources are lowering the required expertise (Herrero‐Huerta *et al*., 2022; Ghosh *et al*., 2023). However, the most critical step is fostering deliberate interdisciplinary collaboration by embedding X‐ray CT specialists within plant–microbe research programmes and ensuring that biological questions, not imaging capabilities, drive experimental design.

With these considerations, X‐ray CT is not a replacement for how we currently conduct plant–microbe experiments. Rather, we propose that it should complement, through interdisciplinary collaborations, the many adjacent approaches that resolve chemical gradients, metabolite exchange, volatile signalling, and other physiological processes central to plant–microbe interactions. X‐ray CT should be viewed as a structural counterpart within a broader experimental toolbox, providing the physical context that enhances, not substitutes, these biochemical and molecular perspectives.

## Conclusions and future directions

Plant–microbe interactions are fundamentally spatial, yet much of the field still uses experimental designs that under‐represent the physical environments in which these interactions occur. This gap between molecular resolution and ecological realism persists largely due to the difficulty of measuring soil and rhizosphere structure. We propose that X‐ray CT will not just reduce this gap; it will allow ambitious hypotheses to be tested regarding a plethora of concurrent plant–microbe interactions in natural soils over time.

Three overarching conclusions emerge. First, structure must be treated as a mechanistic variable. RSA, pore connectivity, aggregate organisation, and root–soil contact actively regulate microbial dispersal, resource availability, infection pathways, and chemical gradients. X‐ray CT captures these features in 3D and over time in intact systems, allowing studies to move beyond bulk descriptors towards spatially explicit hypotheses.

Second, the value of X‐ray CT lies in integration, not in imaging alone. Structural traits acquire biological meaning only when analysed alongside molecular, microbiome, and physiological measurements, and in the context of organismal biology. When X‐ray CT guides sampling, coregistration with omics, or structure‐informed modelling, it provides the spatial scaffold needed to interpret biological data mechanistically.

Third, digital rhizospheres offer a pathway from description to prediction. By combining X‐ray CT‐derived geometry with molecular datasets and mechanistic models, it will become possible to construct system‐specific, structure‐aware representations of plant–soil–microbe interactions. These frameworks enable explicit testing of how root traits, soil management, or microbial communities reshape habitats and influence plant performance and disease outcomes.

Four future priorities will allow X‐ray CT to maximise potential in plant–microbe research:

1. *Design experiments around structure from the outset*. Plant–microbe studies should define spatial and temporal resolution requirements during experimental design rather than retrospectively exploring them. Sampling strategies, inoculation schemes, and molecular analyses can be aligned with X‐ray CT‐defined regions and time points.
2. *Standardise reporting and trait definitions*. Field‐wide guidelines for scan parameters, radiation dose, segmentation workflows, and core properties are needed to ensure comparability. Convergence on a set of biologically interpretable structural traits would enable cross‐study synthesis. Equally, open access to datasets will provide the bedrock for the aspirations of this article.
3. *Treat structure as a first‐class variable*. X‐ray CT‐derived descriptors should be incorporated directly into statistical and mechanistic models. This approach separates structure‐mediated effects from biological responses, improving causal inference and predictive accuracy.
4. *Translate structure‐aware insights into practice*. X‐ray CT‐informed approaches have clear value for soil‐borne disease diagnostics, enabling nondestructive detection and spatial quantification of pathogens in intact soils (Pereira *et al*., 2025). Beyond detection, comparative analysis of pathogen‐positive and pathogen‐negative soils could identify structural features, such as RSAs, pore connectivity, or root–soil contact patterns, that are consistently associated with disease development. These structure–disease relationships provide a mechanistic basis for informing land management strategies, including soil physical interventions and cropping practices that disrupt pathogen‐favourable microhabitats.

In summary, X‐ray CT has already transformed how we visualise roots and soils. Although X‐ray CT is becoming increasingly practical and comparatively accessible, particularly via shared facilities, its routine use is not yet universal, and barriers relating to cost, expertise, and data processing constrain its usage. Its great potential lies in encouraging the field to conceptualise plant–microbe interactions not only by genes and signals but also as processes governed by the three‐dimensional architectures that determine where, when, and how those signals operate. Embedding structure within experimental and theoretical frameworks will be central to building a predictive, physiology‐centred understanding of plant–microbe systems in realistic environments.

## Competing interests

None declared.

## Disclaimer

The New Phytologist Foundation remains neutral with regard to jurisdictional claims in maps and in any institutional affiliations.
